# Current practice in benzodiazepine receptor agonists deprescribing on acute geriatric wards: a cohort study

**DOI:** 10.1186/s12877-022-02753-w

**Published:** 2022-02-01

**Authors:** François-Xavier Sibille, Anne Spinewine, Lorène Zerah, Laurentine Maljean, Didier Schoevaerdts, Marie de Saint-Hubert

**Affiliations:** 1 Department of Geriatric Medicine, CHU Dinant Godinne UCL Namur, Avenue Dr Gaston Therasse, 1, 5530 Yvoir, Belgium; 2 Institute of Health and Society, Université Catholique de Louvain, Clos Chapelle aux Champs, 30 Bte B1. 30.13, 1200 Brussels, Belgium; 3 Clinical Pharmacy Research Group, Louvain Drug Research Institute, Université Catholique de Louvain, Avenue Mounier, 72 bte B1.72.02, 1200 Brussels, Belgium; 4 Department of Pharmacy, CHU Dinant Godinne UCL Namur, Avenue Dr Gaston Therasse, 1, 5530 Yvoir, Belgium; 5 NARILIS, Namur, Belgium

**Keywords:** Benzodiazepines, Deprescribing, Frail elderly, Hospitalization

## Abstract

**Background:**

Benzodiazepine receptor agonist (BZRA) use is highly prevalent in hospitalised older people although these drugs are associated with numerous and serious adverse events. Deprescribing can reduce risks associated with chronic BZRA use. The aim of this study was to measure the prevalence of, and factors associated with, BZRA deprescribing in acute geriatric units.

**Methods:**

During a one-year period, this multicentre retrospective study included patients aged ≥70 years, hospitalised in acute geriatric units, and using ≥1 BZRA on admission. BZRA deprescribing at discharge was defined as: ≥25% decrease in lorazepam-equivalent admission dose; discontinuation of all BZRAs; or cessation of a rescue prescription at discharge. BZRA cessation was defined as discontinuation of all BZRAs at discharge. We identified social, medical, geriatric and medication factors associated with BZRA deprescribing using logistic regression.

**Results:**

In total, 561 patients were included (mean age: 85.3±5.9 years, 70% of women). BZRA deprescribing occurred in 240 (42.8%), including 85 with BZRA cessation (15.2%). Deprescribing occurred more frequently in patients with a BZRA-related adverse event on admission or during hospital stay (odds ratio (OR) 4.5; 95% confidence interval [2.6; 7.9]), with an antidepressant (1.6 [1.1; 2.4]) and a higher lorazepam-equivalent dosage on admission (OR 1.2 [1; 1.4]), and less frequently in patients with antipsychotic drug (OR 0.5 [0.3; 0.8]). BZRA cessation was more likely in patients with a BZRA-related adverse event (OR 2.2 [1.2; 4.3]) and a lower lorazepam-equivalent dosage on admission (OR 0.5 [0.3; 0.6]).

**Conclusions:**

During hospitalisation in the acute geriatric units of our hospital, BZRA deprescribing occurred in 42.8% of the patients. Identification of an BZRA-related adverse event by the treating physician appears to be a major factor: this reactive deprescribing accounted for 74% of cases in our study. Further prospective studies are needed to measure long-term persistence of in-hospital deprescribing and encourage proactive management.

## Introduction

Benzodiazepine receptor agonists (BZRA), including benzodiazepines and z-drugs, are the most prescribed psychotropic drugs in older people [1, 2], with prescription rates ranging from 7% to 25% in elderly persons living in the community [3, 4], to 52% in long-term-care residents [5], and 32.6% in hospitalised patients [6].

BZRAs may be appropriate in a few circumstances, such as seizure disorders or ethanol withdrawal. However, they are mainly used in the treatment of sleep disorders and anxiety [7]. For these indications, they are often inappropriate because of their moderate and short-term efficacy and their numerous and serious adverse events, such as delirium, falls and fractures [8–10]. Although, the American Geriatric Society [11], the START-STOPP criteria [12], and the FORTA list [13] recommend that BZRAs should be avoided in all older people, and certainly in those with dementia, risk of delirium, or risk of falls.

Deprescribing can be defined as the process of withdrawal of an inappropriate medication under the supervision of a healthcare professional with the goal of managing polypharmacy and improving outcomes [14]. There is a growing body of evidence that BZRA deprescribing can reduce the risk of harm associated with chronic BZRA use, with no worsening of, or even improvement in, sleep quality, and reduction in anxiety or depression [15–18]. The success rates reported in older people vary according to the definition of deprescribing (discontinuation only or dose reduction), the setting in which the effects of deprescribing are measured, the nature of the interventions, the type of analysis, and the follow-up duration. For example, BZRA deprescribing success rates range from 27% to 80% in older people living in the community [19–21], and from 33% to 66% in nursing-home settings [5, 22].

Despite this available literature, several research gaps remain. Firstly, few studies have explored BZRA deprescribing during acute hospitalisation. Two studies targeted geriatric units, but were monocentre, had only limited sample sizes, and mental and cognitive disorders were exclusion criteria [23, 24]. Moreover, although there is a global awareness of the barriers and facilitators of successful general deprescribing at the physician (existence of recommendations, concerns about adverse withdrawal effects, medication initially prescribed by another physician … ) and patient (trust in the treating physician, experience of burden from medications, increased involvement … ) levels [25–28], specific factors related to BZRA deprescribing have been less explored. Finally, although geriatricians are well aware of the risks of BZRA use in frail hospitalised older patients, little is known about how much they modify BZRA prescription during usual care in acute geriatric units (AGU).

The main aim of the present study was to measure the prevalence of, and factors associated with, BZRA deprescribing in acute geriatric wards. Secondary aims were to analyse BZRA cessation and associated factors; switch to another BZRA molecule; switch to another sedative molecule.

## Methodology

### Study design, study setting and eligibility criteria

We retrospectively screened the medical records of all patients aged 70 years or older, hospitalised during 2018 in three acute geriatric units (AGUs) in the region of Namur, Belgium. The three AGUs had a combined total of 81 beds and 1435 hospitalisations during 2018, comprising 1285 different patients. The AGUs receive patients with acute pathologies and offer interdisciplinary care (physiotherapist, occupational therapist, speech therapist, and dietician). One AGU is in a teaching hospital and has a clinical pharmacist in the interdisciplinary team.

Patients were included if they were using BZRAs (≥1 BZRA at admission, on a regular basis or as needed). Patients who died during their hospital stay or for whom there was no record of their discharge treatment were excluded because BZRA use at discharge could not be analysed. For patients hospitalised several times during 2018, only the first admission was analysed.

### Outcomes

The main outcome measure was BZRA deprescribing at discharge*,* defined as: ≥25% decrease in lorazepam-equivalent admission dose; discontinuation of all BZRA; or cessation of a rescue BZRA prescription [5]. Secondary outcomes were BZRA cessation at discharge defined as cessation of all BZRAs (subset of BZRA deprescribing), BZRA switch defined as a change of BZRA molecule between admission and discharge with or without a change in lorazepam-equivalent dose, and switch to another sedative molecule defined as new prescriptions of trazodone or mirtazapine at discharge.

### Data collection and variables

All data (demographic, clinical and medication) were extracted from the patient electronic records, including emergency department or other original ward discharge letters, geriatric ward discharge letters and medication lists, geriatric mobile unit reports, and laboratory values. Data extraction was performed by two researchers (a geriatrician, FXS, and a clinical pharmacist with clinical practice in geriatrics, LM) using a written protocol. For a selection of 30 files, extraction was performed independently by both researchers and discrepancies were discussed and clarifications added to the standard operating procedure.

We used the Cumulative Illness Rating Scale-Geriatric (CIRS-G) [29] to estimate comorbidity burden (theoretical maximum score = 56). We recorded the presence or absence of: delirium, according to the clinical judgment of the treating geriatrician; cognitive decline, if a diagnosis of dementia had been established or if the Mini Mental State Evaluation was less than 24 points, outside a context of delirium. Discharge to another ward was defined as discharge to a short stay in a nursing home or to another hospital ward.

On admission and at discharge we collected the number of prescribed medications; the number, molecules and dosages of BZRA (Appendix 1); and the number and molecules of other psychotropic drugs (N02A, N03A, N04, N05A, N05C, N06A-C-D). BZRA dosages were converted into lorazepam-equivalent doses using a conversion table [30, 31], so that molecules could be compared at baseline and between admission and discharge. Polypharmacy was defined as regular use of 5 or more drugs [32] and excessive polypharmacy as use of 10 or more drugs [33]. Multi-BZRA users were defined as users of ≥2 BZRA on a regular basis and/or for rescue use. Psychotropic polypharmacy was defined as concomitant use of ≥3 central nervous system (CNS)-acting drugs [11]. BZRA-related adverse event was defined as any adverse event present on admission or during hospitalization, explicitly related to BZRA use, and documented as such in the electronic medical record, based on the clinical judgment of the treating team. These adverse events were falls, dizziness, drowsiness and delirium.

### Statistical analysis

Data are presented as mean (standard deviation [SD]) or median (first and third quartile [Q1; Q3]) for continuous variables, and number (percentage) for categorical variables. Normality was assessed using the Kolmogorov-Smirnov test and a graphical representation of the distribution. The Mann-Whitney U test or Student t test was used for continuous variables and Pearson’s chi-squared test or Fisher’s exact test for categorical variables.

Binary logistic regression models were developed to assess independent variables associated with BZRA deprescribing and cessation, and adjusted odds ratios (OR) were calculated with their 95% confidence intervals (95CI). Factors potentially associated with BZRA deprescribing were selected through literature review [34–38], included demographic and administrative data (including length of stay), factors related to possible indications for BZRA prescription, comorbidities of relevance when considering BZRA use in older people, and adverse events related to BZRA use (Table 1). Variables with a *p-value* <.10 in univariate analysis (Appendix 4) were eligible for the multivariable model. Correlation between variables was assessed using a principal component analysis. The choice between two correlated variables was made based on their respective clinical relevance. We also included other variables that we expected to be associated with the likelihood of BZRA deprescribing. There were no missing data. All tests were 2-sided, and a *p* < .05 was considered statistically significant.

**Table 1:** Population characteristics

	BZRA users ***N***=561	BZRA deprescribing ***N***= 240	BZRA continuation ***N***= 321	***p*** value^**a**^
**Demographic data**				
Age (years), mean, SD	85.3; 5.9	84.8; 5.9	85.7; 5.8	.066
Female, n (%)	392 (69.9)	168 (70.0)	224 (69.8)	.999
Place of residence, n (%)				
Home	412 (73.4)	165 (68.7)	247 (76.9)	
Nursing home	149 (26.6)	75 (31.3)	74 (23.1)	**.015**
**Comorbidities**				
CIRS-G, median; Q1-Q3	22; 18-25	22; 19-26	21; 17-25	**<.001**
Anxiety, n (%)	92 (16.4)	44 (18.3)	48 (15.0)	.340
Sleep disorder, n (%)	49 (8.7)	22 (9.2)	27 (8.4)	.871
Restless leg, n (%)	16 (2.9)	7 (2.9)	9 (2.8)	.999
Depression, n (%)	149 (26.6)	71 (29.6)	78 (24.3)	.192
Bipolar or psychotic disorder, n (%)	16 (2.9)	7 (2.9)	9 (2.8)	.999
Extrapyramidal syndrome, n (%)	58 (10.3)	21 (8.8)	37 (11.5)	.353
Previous fracture, n (%)	201 (35.8)	93 (38.8)	108 (33.6)	.247
Fall at admission, n (%)	274 (48.8)	115 (47.9)	159 (49.5)	.769
Current fracture, n (%)	85 (15.2)	31 (12.9)	54 (16.8)	.247
COPD, n (%)	82 (14.6)	36 (15.0)	46 (14.3)	.919
Delirium, n (%)	188 (33.5)	95 (39.6)	93 (29.0)	.017
Cognitive decline, n (%)	316 (56.3)	148 (61.7)	168 (52.3)	.034
**Administrative data**				
Admission, n (%)				
Emergency	110 (19.6)	52 (21.7)	58 (18.1)	
Transfer	65 (11.6)	34 (14.2)	31 (9.7)	.099
Direct to AGU	386 (68.8)	154 (64.2)	232 (72.3)	
Length of stay (days), median; Q1-Q3	15; 11-22	16; 12-23	15; 11-22	.181
Discharge destination, n (%)				
Home	277 (49.4)	107 (44.6)	170 (53.0)	
Nursing home	200 (35.7)	102 (42.5)	98 (30.5)	**.040**
Another ward	84 (14.9)	31 (12.9)	53 (16.5)	
**Medication at admission**				
Total medications, median; Q1-Q3	9; 7-12	9; 7-12	9; 7-11	.174
Polypharmacy, n (%)	514 (91.6)	218 (90.8)	296 (92.2)	.668
Excessive polypharmacy, n (%)	250 (44.6)	116 (48.3)	134 (41.7)	.142
BZRA dose, median; Q1-Q3	1.0; 1.0- 2.0	1.33; 0.9-2.5	1.0; 1.0-2.0	**.023**
Multi-BZRA users, n (%)	112 (20.0)	73 (30.4)	39 (12.1)	**<.001**
Psychotropic polpharmacy, n (%)	231 (41.2)	119 (49.6)	112 (34.9)	**<.001**
Antipsychotic users, n (%)	116 (20.7)	43 (17.9)	73 (22.7)	.270
Antidepressant users, n (%)	273 (48.7)	135 (56.3)	138 (43.0)	**.002**
Trazodone	59 (10.5)	32 (13.3)	27 (8.4)	.054
Mirtazapine	45 (8.0)	21 (8.8)	24 (7.5)	.586
**Miscellaneous**				
Palliative status, n (%)	42 (7.5)	20 (8.3)	22 (6.9)	.619
Intervention of a clinical pharmacist, n (%)	202 (36.0)	103 (42.9)	99 (30.8)	**.002**
Identification of a BZRA-related adverse event by the treating physician, n (%)	88 (15.7)	65 (27.1)	23 (7.2)	**<.001**
BZRA switch between admission and discharge, n (%)	65 (11.6)	26 (10.8)	39 (12.1)	.870

*SD* standard deviation, *Q1* first quartile, *Q3* third quartile, *CIRS-G* Cumulative Illness Rating Scale-Geriatric, *COPD* Chronic Obstructive Pulmonary Disease, *AGU* acute geriatric unit

^a^ *p* value of difference between deprescribing and continuation using Mann-Whitney U test or Student t test for continuous variables and Pearson’s chi-squared test or Fisher’s exact test for categorical variables

All analyses were performed using R software v.4.0.3 (R Foundation for Statistical Computing, Vienna, Austria).

## Results

### Population description

**Fig. 1:**
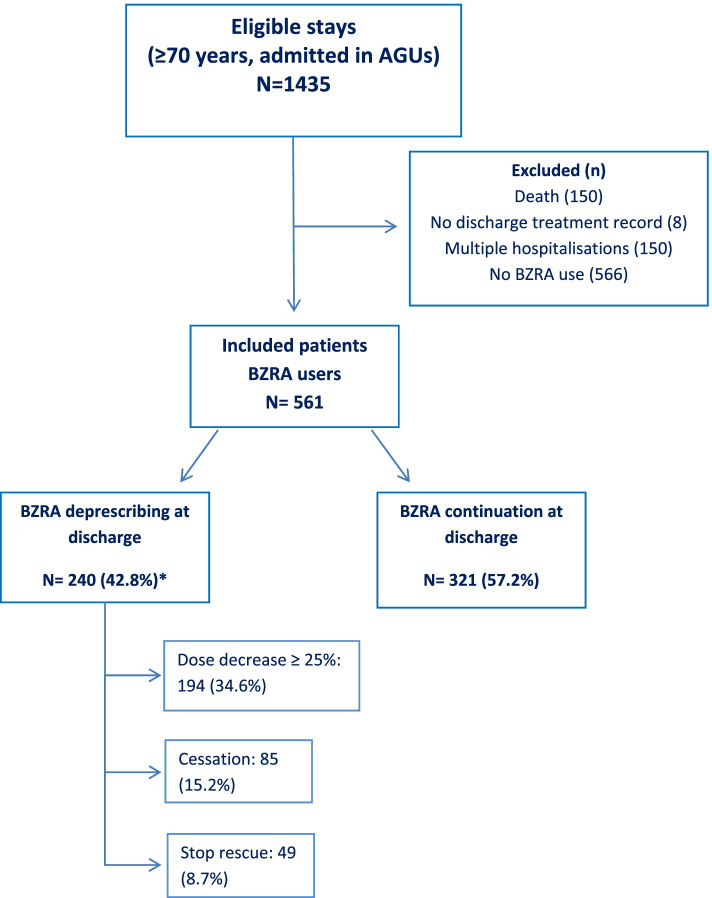
flow chart. AGUs: acute geriatric unit. * Patients may have either decrease or discontinuation of a daily-used molecule, and cessation of a rescue prescription

Among the 1285 patients admitted in AGUs, 561 were using BZRA and were included in the study (Figure 1). Patient characteristics are shown in Table 1. The mean patient age was 85.3±5.9 years; 70% were women and 73% lived in the community. The level of comorbidities was high (median CIRS-G 22 points) [39]. Anxiety and sleep disorders were mentioned for 16.4% and 8.7% of patients, respectively. More than half of the patients had cognitive decline and one third had delirium on admission. Polypharmacy and excessive polypharmacy were highly prevalent: 91.6% of the patients were taking 5 or more daily medications and 44.6% 10 or more. Psychotropic polypharmacy affected 41.2% of the patients (Table 1). Median length of stay was 15 days with (interquartile range: 11-22 days). Patient characteristics by AGU are presented in Appendix 2.

### BZRA deprescribing and associated factors

At discharge, BZRAs had been deprescribed in 240 patients (42.8%): 85 patients (15.2% of all included patients) had stopped taking any BZRA (i.e. BZRA cessation), 194 (34.6%) had had a reduction in lorazepam-equivalent dosage of at least 25%, and 49 (8.7%) had stopped taking one or more rescue BZRAs. In some patients two of the three criteria were met.

In the multivariate analysis (Table 2), a BZRA-related adverse event during the hospital stay (OR 4.5 [2.6 -7.9]), a higher lorazepam-equivalent dose (OR 1.2 [1-1.4]) and use of an antidepressant (1.6 [1.1; 2.4]) were significantly associated with BZRA deprescribing. In the opposite, patients using also an antipsychotic drug on admission had a significantly lower rate of BZRA deprescribing (OR 0.5 [0.3-0.8]).

**Table 2:** Factors associated with BZRA deprescribing and BZRA cessation: multivariate analysis

	BZRA deprescribing		BZRA cessation	
	OR [95CI]	*p* value	OR [95CI]	*p* value
**Demographic data**				
Age^a^	1 [0.9;1.0]	.218	1 [0.9;1.0]	.085
**Comorbidities**				
CIRS-G^a^	1 [1;1.1]	.110	1 [1.0;1.1]	.489
Depression	1.2 [0.8;1.9]	.365	0.9 [0.5;1.7]	.809
Delirium	1.2 [0.8;1.8]	.457	1.2 [0.7;2.1]	.474
Cognitive decline	1.5 [1;2.3]	.066	1 [0.6;1.8]	.971
Anxiety	1.2 [0.7;2.1]	.437	1 [0.5;2]	.991
Sleep disorder	1.1 [0.6;2.1]	.780	1.7 [0.7;3.7]	.191
**Administrative data**				
Admission directly to AGU	1		1	
Admission through emergency room	1.1 [0.7; 1.9]	.651	0.7 [0.3;1.3]	.256
Admission from another ward	1.5 [0.8; 2.6]	.193	1 [0.4;2]	.931
Length of stay^a^	1 [1;1]	.215	1 [1;1]	.116
Living in a nursing home	1.3 [0.7;2.3]	.592	1.4 [0.6;3.2]	.527
Discharge to home	1		1	
Discharge to a nursing home	1.5 [0.8;2.7]	.172	1.4 [0.6;2.9]	.387
Discharge to another ward	0.8 [0.4;1.4]	.404	0.5 [0.2;1.1]	.124
**Medications at admission**				
BZRA dosage^a^	1.2 [1.0;1.4]	.020	0.5 [0.4;0.6]	<.001
Antipsychotic user	0.5 [0.3;0.8]	.008	0.7 [0.3;1.3]	.236
Antidepressant user	1.6 [1.1;2.4]	.027	1.4 [0.8;2.5]	.243
**Miscellaneous**				
Intervention of a clinical pharmacist	1.5 [0.9;2.3]	.162	0.8 [0.4;1.5]	.421
Identification of a BZRA-related adverse event by the treating physician	4.5 [2.6;8]	<.001	2.4 [1.2;4.5]	.012

*CIRS-G* Cumulative Illness Rating Scale-Geriatric, *OR* Odds ratio, *95CI* 95% confidence interval

^a^Numeric variables

### BZRA cessation and associated factors

At discharge, 85 patients (15.2%) were no longer prescribed any BZRA. In the multivariate analysis (Table 2), BZRA-related adverse event during the hospital stay (OR 2.2 [1.2; 4.3]) was significantly associated with BZRA cessation. Patients with higher lorazepam-equivalent dosage on admission experienced significantly less BZRA cessation (OR 0.5 [0.3; 0.6]).

### Switch of BZRA molecule

The most frequent BZRA molecules on admission were lorazepam (30.2%) and alprazolam (14.7%) for regular users, and alprazolam (31.4%) and prazepam (27.9%) for rescue users (Appendix 3). Regarding the half-lives of the molecules, 70.8% of the patients were taking a medium-acting BZRA. At discharge, the most frequent molecules were lorazepam and alprazolam for both regular and rescue use. BZRA switch occurred in 11.6% of patients. There was a significant shift from long- to medium-acting molecules at discharge (76.7% for regular use and 89.3% for rescue use, *p*=0.025 and *p*<0.001, respectively) (Appendix 3). There were no differences between groups with or without BZRA deprescribing.

### Switch to another sedative molecule

Fourteen (6.0%) of the patients who had BZRA deprescribing were given new prescriptions for trazodone, and 6 (1.8%) of the patients with BZRA continuation (*p*=0.011). Mirtazapine was newly prescribed to 27 patients, distributed evenly between the groups with and without deprescribing (data not shown). Eight patients in the BRZA cessation group (9.4%) were given new prescriptions for trazodone, and 12 (2.5%) in the patients with BRZA continuation (*p*=0.006). There were no differences in new mirtazapine prescriptions in the patients with BZRA cessation and BZRA continuation.

## Discussion

At discharge, respectively 42.8% and 15.2% of older patients hospitalized in 3 Belgian AGUs experienced BZRA deprescribing and cessation. The presence of a BZRA-related adverse event on admission or during hospitalisation was the main predictor of both outcomes. This deprescribing in response to an adverse clinical trigger is called reactive deprescribing and accounted for 74% of deprescribing occurrences in our study. During hospitalisation, there was also a significant switch from long- to medium-acting molecules at discharge. Respectively 12.0% and 14.1% of the patients with BZRA deprescribing or cessation were prescribed another sedative molecule. To the best of our knowledge, this is the first complete description of BZRA deprescribing (including dose tapering and psychotropic drugs co-prescription) during hospitalisation in AGUs.

Our rate of deprescribing is lower than the 53% of patients with a fall in a geriatric rehabilitation context in Australia [40]. Our rate of cessation (15.2%) was also lower than the rate (20.8%) of the control group in the pilot study that evaluated the efficacy of a patient- educational brochure [41]. Similar to our data, an English observational study found that reactive deprescribing accounted for 80% of all drugs combined deprescribing activities during acute hospitalisation [42].

Higher BZRA doses significantly increased the probability of BZRA deprescribing but reduced the probability of BZRA cessation. For any increase of one unit lorazepam-equivalent, there was 20% more chance of BZRA deprescribing and 50% less chance of BZRA cessation. We can hypothesise that this contrasting finding may be the result of the limited length of stay of our patients. Most BZRA tapering methods recommend schedules of at least one month [41], but three quarters of our patients had a length of stay of less than 22 days. Use of an antidepressant was associated with a higher probability of BZRA deprescribing, in line with what was suggested by a recent Cochrane Database of Systematic Review [43]. Use of an antipsychotic was associated with a lower probability of BZRA deprescribing, which has not, to our knowledge, been described previously. We can hypothesize that patients using antipsychotic drugs may have more severe behavioural symptoms.

We expected the intervention of a clinical pharmacist to influence BZRA deprescribing and cessation. Indeed, several trials using pharmacist involvement have shown a positive impact on BZRA deprescribing [44–46]. A recent systematic review suggested that pharmacist involvement as part of a multidisciplinary team might improve the quality of prescribing in older inpatients [47]. Despite greater deprescribing in the univariate analysis with intervention of a clinical pharmacist, this was not confirmed in the multivariable analysis, possibly due to insufficient power and because part of this effect was captured by the BZRA-related adverse events, which a clinical pharmacist may help to identify.

One may be concerned that BZRA deprescribing would just reflect a switch to another sedative medication, such as trazodone or mirtazapine [48, 49]. Indeed, psychotropic polypharmacy was already high in our population [6] and is also a subject of concern in older people [11]. It is encouraging to see that this situation did not occur frequently, as another sedative molecule was prescribed in only 12.0% and 14.1% of BZRA deprescribing or cessation cases.

In patients without BZRA deprescribing, BZRA molecule was switched in 11.6% of cases, with a significant switch from long- to medium-acting molecules with a better pharmacokinetic profile in older people. This change in profile may be viewed as an improvement [50].

One aspect regarding our population deserves closer examination: anxiety and sleep disorders were rarely mentioned in the medical records despite high general comorbidity rates [51]. This low rate of anxiety and sleep disorders suggests that BZRAs may not be indicated in these patients [5], and/or that the indication for BZRA is often not documented. Identifying the indication for a medication is the first step in developing a deprescribing algorithm [52], and is an important challenge and an area that needs to be improved to promote deprescribing.

Hospitalisation may be questioned as the best setting for deprescribing of such medication. Indeed, in line of the results of the present study, it represents an opportunity to initiate deprescribing for several reasons [42, 48]: 1) Patients can benefit from close monitoring of withdrawal symptoms; 2) frequent diagnoses on admission, such as falls or delirium, may be related to BZRA use before hospitalisation; 3) AGUs provide patient-centred approach, multidisciplinary teams and geriatrician insight, all known to improve deprescribing rates [5, 18, 34, 53]. However, hospitalised patients may experience more anxiety and sleep disorders [51], and have less confidence in healthcare professionals at the hospital than in their general practitioner or usual nursing team. The acute medical problem leading to hospitalisation may also detract the attention of the professionals away from performing BZRA deprescribing. Recent data inform on key determinants and behavioural change techniques to improve deprescribing in the hospital setting [54]. For instance, interventions should facilitate shared deprescribing decision making.

The strength of this study is that it provides a real-life picture of BZRA management in the AGUs. No specific deprescribing program was implemented and patients received usual geriatric care. We also included cognitively impaired patients who are often excluded from deprescribing trials [21, 55], and provide a context of other psychotropic drugs use. The main limitations of the study are its retrospective design limiting the availability and quality of the data. For example, no systematic screening for delirium was performed, so that its prevalence may have been underestimated, especially that of hypoactive delirium. Secondly, because of the lack of follow-up, we do not know whether BZRA deprescribing was maintained or whether BRZAs were started again after hospital discharge [48, 56]. We also have no information on the duration of BZRA use, alcohol consumption, and deprescribing failure or refusal during the hospital stay, all factors that could potentially influence BZRA deprescribing rates.

In conclusion, this retrospective study showed that BZRA deprescribing was initiated in 42.8% of patients hospitalised in 3 Belgian acute geriatric wards. Identification of a BZRA-related adverse event by the treating physician, higher lorazepam-equivalent dosage and use of an antidepressant were associated with BZRA deprescribing and cessation. Use of an antipsychotic drug was associated with a lower rate of BZRA deprescribing. Routine clinical work should better document indications for BZRA intake and identify opportunities to proactive deprescribing. Future studies should investigate mid-term persistence of BZRA deprescribing and evaluate patient- and healthcare professional-centred interventions to optimise BZRA deprescribing in hospital.

## Data Availability

The datasets used and/or analysed during the current study are available from the corresponding author on reasonable request.

## Appendix 1

Tables 3

**Table 3:** BZRA lorazepam-equivalent dosage and duration of action

INN	ATC code	Equivalent dosage (mg)	Duration of action
diazepam	N05BA01	10	Long-acting
alprazolam	N05BA12	0.5	Medium-acting
bromazepam	N05BA08	4.5	Medium-acting
brotizolam	N05CD09	0.25	Medium-acting
clobazam	N05BA09	10	Long-acting
clonazepam	N03AE01	0.5	Long-acting
clorazepate	N05BA05	10	Long-acting
clotiazepam	N05BA21	5	Medium-acting
cloxazolam	N05BA22	1	Long-acting
flunitrazepam	N05CD03	0.5	Long-acting
flurazepam	N05CD01	15	l Long-acting
ethyl loflazepate	N05BA18	1	Long-acting
loprazolam	N05CD11	0.5	Medium-acting
lorazepam	N05BA06	1	Medium-acting
lormetazepam	N05CD06	1	Medium-acting
midazolam	N05CD08	7.5	Short-acting
nitrazepam	N05CD02	5	Long-acting
nordazepam	N05BA16	2.5	Long-acting
oxazepam	N05BA04	15	Medium-acting
prazepam	N05BA11	20	Long-acting
triazolam	N05CD05	0.125	Short-acting
zolpidem	N05CF02	10	Short-acting
zopiclone	N05CF01	7.5	Short-acting

*INN* international non-proprietary name, *ATC* Anatomic Therapeutic Chemical classification

## Appendix 2

Table 4

**Table 4:** Patients’ characteristics by AGU

	AGU 1 (*n*= 172)	AGU 2 (*n*= 215)	AGU 3 (*n*= 174)
**Demographic data**			
Age (years): mean; SD	86.7; 5.6	84.4; 5.7	85.1; 6.2
Female	123 (71.5%)	143 (66.5%)	126 (72.4%)
**Comorbidities**			
CIRS-G: median; Q1-Q3	20; 17-23	24; 20-28	20.5; 17-24.75
Anxiety	10 (5.8%)	59 (27.4%)	23 (13.2%)
Sleep disorder	5 (2.9%)	26 (12.1%)	18 (10.3%)
Restless leg syndrome	1 (0.6%)	11 (5.1%)	4 (2.3%)
Depression	33 (19.2%)	63 (29.3%)	53 (30.5%)
Extrapyramidal syndrome	13 (7.6%)	25 (11.6%)	20 (11.5%)
COPD	23 (13.4%)	34 (15.8%)	25 (14.4%)
Cognitive decline	129 (75.0%)	83 (38.6%)	104 (59.8%)
Delirium	55 (32.0%)	76 (35.3%)	57 (32.8%)
Fall at admission	77 (44.8%)	112 (52.1%)	85 (48.9%)
Current fracture	22 (12.8%)	30 (14.0%)	33 (19.0%)
Previous fracture	60 (34.9%)	83 (38.6%)	58 (33.3%)
Psychiatric disorder	3 (1.7%)	5 (2.3%)	8 (4.6%)
**Administrative data**			
Place of residence:			
Home	122 (70.9%)	169 (78.6%)	121 (69.5%)
Nursing home	50 (29.1%)	46 (21.4%)	53 (30.5%)
Length of stay (days): median; Q1-Q3	15; 11.75-20	15; 11-21.5	16; 11-26
Admission route:			
Emergency	146 (84.9%)	116 (54.0%)	124 (71.3%)
Direct	15 (8.7%)	70 (32.6%)	25 (14.4%)
Transfer	11 (6.4%)	29 (13.5%)	25 (14.4%)
Discharge destination:			
Home	88 (51.2%)	108 (50.2%)	81 (46.6%)
Nursing home	61 (35.5%)	68 (31.6%)	71 (40.8%)
Another ward	23 (13.4%)	39 (18.1%)	22 (12.6)
**Medications at admission**			
Total medications: median; Q1-Q3	8.5; 6-10.25	10; 8-13	8; 6-11
Polypharmacy	149 (86.6%)	206 (95.8%)	159 (91.4%)
Excessive polypharmacy	63 (36.6%)	122 (56.7%)	65 (37.4%)
BZRA dose: median; Q1-Q3	1.3; 1-2.5	1; 0.95-2	1; 0.85-2
Multi-BZRA users	29 (16.9%)	51 (23.7%)	32 (1.8%)
Psychotropic drug users	119 (69.2%)	157 (73.0%)	123 (70.7%)
Psychotropic polypharmacy	69 (40.1%)	97 (45.1%)	65 (37.4%)
Antipsychotic users	39 (22.7%)	43 (20.0%)	34 (19.5%)
Antidepressant users	76 (44.2%)	118 (54.9%)	79 (45.4%)
Trazodone users	13 (7.6%)	30 (14.0%)	16 (9.2%)
**Miscellaneous**			
Palliative status	10 (5.8%)	8 (3.7%)	24 (13.8%)
Intervention of a clinical pharmacist	0 (0.0%)	202 (94.0%)	0 (0.0%)
Identification of a BZRA-related adverse event by the treating physician	11 (6.4%)	51 (23.7%)	26 (14.9%)
BZRA switch between admission and discharge	11 (6.4%)	22 (10.2%)	32 (18.4%)

*SD* standard deviation, *Q1* first quartile, *Q3* third quartile, *CIRS-G* Cumulative Illness Rating Scale-Geriatric, *COPD* Chronic Obstructive Pulmonary Disease, *AGU* acute geriatric unit

## Appendix 3

Table 5

**Table 5:** BZRA molecules and categories of duration of action

	Admission	Discharge	*p* value
**Regular use**			
*BZRA frequent molecules*			
Alprazolam	101 [14.7%]	88 [15.6%]	<.001
Bromazepam	63 [9.1%]	46 [8.2%]	
Lorazepam	208 [30.2%]	251 [44.6%]	
Lormetazepam	73 [10.6%]	65 [11.5%]	
Zolpidem	71 [10.3%]	55 [9.8%]	
*BZRA categories*			
Short-acting	91 [13.2%]	75 [12.2%]	.025
Medium-acting	488 [70.8%]	470 [76.7%]	
Long-acting	110 [16.0%]	68 [11.1%]	
**Rescue use**			
*BZRA frequent molecules*			
Alprazolam	27 [31.4%]	18 [23.7%]	.040
Bromazepam	4 [4.7%]	4 [5.3%]	
Lorazepam	15 [17.4%]	43 [56.6%]	
prazepam	24 [27.9%]	3 [3.9%]	
*BZRA categories*			
Short-acting	5 [5.9%]	2 [2.7%]	<.001
Medium-acting	51 [60.0%]	67 [89.3%]	
Long-acting	29 [34.1%]	6 [8.0%]	

## Appendix 4

Table 6

**Table 6:** Factors associated with BZRA deprescribing: Univariate analysis

	OR [95CI]	*p* value
**Demographic data**		
Age	1 [0.9;1]	.080
Female	1 [0.7;1.4]	.927
Place of residence		
Home	1	
Nursing home	1.5 [1;2.2]	.030
**Comorbidities**		
CIRS-G	1.1 [1;1.1]	.001
Anxiety	1.3 [0.9;2.1]	.194
Sleep disorder	1.2 [0.6;2.1]	.636
Restless leg syndrome	1.1 [0.4;3]	.867
Depression	1.3 [0.9;2]	.161
Bipolar or psychotic disorder	1.1 [0.4;3]	.867
Extrapyramidal syndrome	0.7 [0.4;1.1]	.147
Previous fracture	1.3 [0.9;1.8]	.201
Fall at admission	1 [0.7;1.3]	.825
Current fracture	0.8 [0.5;1.2]	.288
COPD	1 [0.6;1.7]	.847
Delirium	1.7 [1.2;2.4]	.009
Cognitive decline	1.5 [1.1;2.1]	.028
**Administrative data**		
Admission		
Direct to AGU	1	
Emergency	1.3 [0.9;2]	.214
Transfer	1.7 [1;2.9]	.033
Length of stay	1 [1;1]	.126
Discharge destination		
Home	1	
Nursing home	1.6 [1.1;2.3]	.007
Another ward	0.9 [0.5;1.4]	.776
**Medication at admission**		
Total medications	1 [1;1.1]	.218
Polypharmacy	0.8 [0.4;1.5]	.460
Excessive polypharmacy	1.3 [1;1.9]	.094
BZRA dose	1.3 [1.1;1.5]	.003
Multi-BZRA users	3.3 [2.2;5.2]	<.001
Psychotropic polpharmacy	2 [1.4;2.8]	<.001
Antipsychotic users	0.8 [0.5;1.2]	.164
Antidepressant users	1.8 [1.3;2.5]	.002
Trazodone	1.8 [1;3]	.033
Mirtazapine	1.2 [0.7; 2.3]	.483
**Miscellaneous**		
Palliative status	1.2 [0.6;2.2]	.630
Intervention of a clinical pharmacist	1.8 [1.2;2.5]	.003
Identification of a BZRA-related adverse event by the treating physician	5.1 [3.1;8.6]	<.001
BZRA switch between admission and discharge	0.9 [0.5; 1.6]	.766

## Abbreviations

AGU: Acute geriatric wards
BZRA: Benzodiazepine receptor agonists
CIRS-G: Cumulative Illness Rating Scale-Geriatric
CNS: Central nervous system
COPD: Chronic Obstructive Pulmonary Disease
OR: Odd’s ratio
Q1; Q3: First and third quartiles
SD: Standard deviation
95CI: 95% confidence intervalle
